# Comparison of the Effect of Synthetic (Tannic Acid) or Natural (Oak Bark Extract) Hydrolysable Tannins Addition on Fatty Acid Profile in the Rumen of Sheep

**DOI:** 10.3390/ani12060699

**Published:** 2022-03-10

**Authors:** Małgorzata P. Majewska, Renata Miltko, Grzegorz Bełżecki, Aneta Kędzierska, Barbara Kowalik

**Affiliations:** The Kielanowski Institute of Animal Physiology and Nutrition, Polish Academy of Sciences, 05-110 Jabłonna, Poland; r.miltko@ifzz.pl (R.M.); g.belzecki@ifzz.pl (G.B.); a.kedzierska@ifzz.pl (A.K.); b.kowalik@ifzz.pl (B.K.)

**Keywords:** oak bark extract, tannic acid, fatty acid, biohydrogenation, rumen, sheep

## Abstract

**Simple Summary:**

Tannins are known as water-soluble polyphenols with the ability to form complexes with macromolecules of dietary and endogenous origin. Thus, they are becoming increasingly popular among scientists as an invaluable “tool” for targeted modification of digestive processes in the rumen. The aim of the study was to compare the effect of tannins of various origin—oak bark extract (natural) vs. tannic acid (synthetic) on the fatty acid composition in the ruminal fluid of sheep. It was shown that both sampling time and animal diet had a significant effect on tested parameters, but this effect was varied. The addition of tannic acid to sheep diet had a greater influence on the fatty acid profile in the rumen than oak bark extract. Differences in the effects of tested additives may arise from the presence of various types of tannins in oak bark extract. Increased concentrations of C18:2 *c9c12* and C18:3 *c9c12c15* in sheep fed diet with tannic acid addition may suggest inhibition of the initial stage of fatty acid biohydrogenation. These promising results may increase knowledge about the action of such compounds on lipid metabolism in the rumen, and provide the basis for further research on health-promoting properties of ruminant products.

**Abstract:**

The aim of the study was to compare two sources of tannins on fatty acids (FA) composition in rumen. Treatments were (g tannins/kg diet as-feed-basis) as follows: (1) no supplemental tannin addition (CON), (2) addition of 13 g of oak bark extract (OAK), and (3) 4 g of tannic acid (TAN). The basal diet contained 55:45 forage to concentrate ratio. Net consumption of tannins (g/d) was 4 g for both tannins sources. The study was performed on three Polish Mountain ewes fitted with rumen cannulas, and was divided into three experimental periods (I, II, and III). Both sampling time and animal diet had a significant effect on FA profile in the rumen fluid. In general, FA concentrations were higher before feeding in comparison to samples collected 2 and 4 h after feeding. In terms of dietary effect, it was shown that TAN addition had a greater influence on FA profile in the ruminal fluid than the OAK diet. Briefly, in the TAN group significantly increased concentrations of C18:2 *c9c12* (linoleic acid, LA) 8 h after feeding (vs. control, CON and OAK), C18:3 *c9c12c15* (α-linolenic acid, LNA) 4 h after feeding (vs. OAK), C20:3 n-6 before feeding (vs. CON), C20:4 before feeding (vs. CON and OAK) and 8 h after feeding (vs. OAK) were recorded. In contrast, OAK addition significantly reduced C20:3 n-6 concentration 2 h after feeding (vs. CON). In conclusion, increased concentrations of both LA and LNA in the rumen indicated that supplemental tannic acid may inhibit the initial stage of FA biohydrogenation in the rumen.

## 1. Introduction

Tannins are a group of water-soluble polyphenols with different chemical structure (mainly of condensed and hydrolysable types) and reactivity, commonly found in the natural environment [1]. They exhibit unique properties, including the ability to form complexes mainly with proteins of various origin (dietary, endogenous, microbiological), but also to a lesser extent with other components, such as metal ions and carbohydrates [2,3]. The formation of complexes with feed ingredients or enzymes results in the protection of nutrients against digestion, or even a significant reduction in their degradation in the rumen [4]. The low pH in the abomasum (below 3.5) promotes the release of complexes with tannins, making it possible to digest the dietary protein and increase absorption of amino acids in the small intestine [5]. Depending on the type of tannins, their fate in the digestive tract is different. Condensed tannins appear to pass intact through the digestive tract. Only those tannins with low polymerization (such as dimers) can be absorbed and enter the bloodstream [6]. The remaining undigested condensed tannins are excreted in the feces of animals [7]. On the other hand, literature data proved that hydrolysable tannins can be degraded by microbial activity [5,7], and their intermediate metabolites (e.g., gallic acid) are found in the urine [8]. 

Tannins are generally considered to be inhibitors of microbial growth [9], and their presence can modulate both quantitative and qualitative composition of microflora and microfauna [10,11]. In recent years, it has been shown that these compounds can also significantly affect lipid metabolism, including fatty acid (FA) biohydrogenation in the rumen [12,13,14]. This process occurs with the participation of ruminal microorganisms and involves a series of isomerizations and reductions of polyunsaturated fatty acids (PUFA), leading to several intermediates, including conjugated linoleic acids (CLA), and, finally, stearic acid (C18:0, SA) formation [15,16]. Therefore, products derived from ruminants are primarily characterized by a high concentration of saturated fatty acids, which are associated with cardiovascular diseases [17]. Previous studies have shown the potential of tannins to modify the process of biohydrogenation in the rumen, but some data are inconsistent, and several mechanisms of tannin action have been proposed. Overall, tannins have been reported to inhibit the final stage of FA biohydrogenation in the rumen, manifested by the accumulation of intermediates and reduced SA production [18,19]. However, some studies have shown a different direction in which tannins may stimulate the early stage of biohydrogenation, thereby increasing the disappearance of C18:2 *c9c12* (linoleic acid, LA) and C18:3 *c9c12c15* (α-linolenic acid, LNA), rather than inhibiting SA production [20,21].

Recently, many in vitro studies exploring the potential of tannins have been conducted, but the number of works involving animals is still insufficient. In the present study, hydrolysable tannins of different origin—natural (oak bark extract, OAK) and synthetic (tannic acid, TAN)—were added to sheep diets. One of the advantages of using oak bark is its universality, as it is a waste of the wood industry [22]. Additionally, as a plant used in medicine, it has desired properties (antioxidant, anti-inflammatory, antibacterial) [23]. Due to the fact that restrictions on the use of antibiotics in animal nutrition have been introduced, the use of natural environment resources, in this case, the native oak species, is highly recommended. In our previous studies, the addition of oak bark with much lower tannin contents, and additionally containing other biologically active compounds, was used [24,25], and it was difficult to interpret results when studying the effect of tannins (as the only factor) on the examined indicators. Thus, in the present study, pure extract, due to the concentrated amount of tannins (30 g in 100 g of the extract), was used. It is considered that presence of polyphenolic compounds (tannins) in OAK may significantly affect microorganisms inhabiting the rumen and indirectly affect processes involved in. In contrast, TAN is a pure chemical compound belonging to gallotannins (hydrolysable tannins). Importantly, it is well known that hydrolysable tannins (including TAN) can be degraded to gallic acid, pyrogallol, and resorcinol, and ultimately to short-chain FA (acetate and butyrate) [8,26], thus, as a consequence, may have an essential impact on rumen parameters. More importantly, the selection of hydrolysable tannins in both additives was determined by their high reactivity with feed components, in comparison to condensed ones [6]. This property is mainly related to the lower molecular weight as well as susceptibility to degradation by several microorganisms inhabiting the rumen [11].

Tannins are known to be anti-nutritional substances. According to Silanikove et al. [27], the concentration of tannins above 3% in ruminants’ diet may reduce the palatability of feed, resulting in depressed feed intake and digestion of nutrients. Hydrolysable tannins are particularly dangerous, due to the fact that they can be decomposed in the rumen to the metabolites of low molecular weight [6], and may exert toxic effect to the liver and kidney in high doses [28]. Thus, in the present study, it was decided the dose of 4 g according to the previous report of Rojas-Román et al. [29] should be used, in which lambs supplemented with 4 g tannins/kg diet showed maximal responses in growth performance, while supplementation over 4 g/kg diet affected negatively the growth performance responses. In our opinion, the low proportion of tannins in the diet will allow animals use the natural adaptive mechanisms and directionally modify physiological processes in the rumen, and, at the same time, present a practical (economical) aspect of using such additives in animal production in the future.

We hypothesized that the tested additives could impair FA biohydrogenation in the rumen, while simultaneously increasing CLA *c9t11* and decreasing SA concentrations. Thus, the aim of the study was to compare the effect of OAK and TAN on FA concentration in the ruminal fluid of sheep. This allowed us to assess whether the effect of tested additives on FA concentration was similar, or whether, the impact was completely different, and then it would be possible to identify the reasons for this. In the case of visible impact of tested additives on FA concentration, the exact mechanism of tannin action could be proposed.

## 2. Materials and Methods

### 2.1. Animals and Diets

For this study, three Polish Mountain ewes (48.7 ± 4.31 kg live weight) fitted with rumen cannulas were used in a 3 × 3 Latin square experimental design with the aim to compare the effects of two sources of tannins on ruminal fatty acid composition. The experimental periods lasted 37-d, and was divided into: 14-d of gradual transition to a diet, 21-d of adaptation to a specific diet, and finally 2-d of sampling. It is worth noting that days of gradual transition to a diet (also known as a ‘silencing period’) were introduced to the experiment during changes of animal diets in subsequent periods of the experiment. Briefly, each sheep initially received a control diet, and only then received another diet assigned to the relevant period. This allowed the ruminal microorganisms to adapt to new environmental conditions and to reduce the possible impact of the diet from previous periods on the final results. The basal diet (CON) offered consisted of meadow hay, barley meal, soybean meal, and vitamin-mineral premix, and was formulated according to mature ewes’ requirements (Table 1). Treatments (g tannins/kg diet as-feed-basis) were as follows: (1) no supplemental tannin addition (CON), (2) addition of 13 g of oak bark extract (OAK, Chem&Pol Sp. z o.o., Warsaw, Poland), and (3) 4 g of tannic acid (TAN, Sigma-Aldrich, Poznań, Poland). Tannins were manually mixed with a concentrate (barley and soybean meal with premix) before mixing with hay, and finally offered to the ewes. Both additives were in the form of powder. Due to the net concentration of each source of tannins (30% of OAK and 100% of TAN), the final net dose of tannin targeted were 4 g/kg diet as feed-basis. Since tannins are anti-nutritional substances, they were introduced into the animals’ diet gradually, starting with a half dose (6.5 g of OAK and 2 g of TAN), and ending with the target amount. To avoid feed refusals during experimental period, the daily feed intake (as-feed-basis) was restricted to 2.10% of the live weight of ewes (equivalent to daily 1.025 kg feed/ewe). Ewes were provided fresh feed twice daily at 7.00 and 15.00 h in equal proportion. The total daily dosage of tannins per ewe was concentrated in the diet provided in the morning feeding (all ewes were fed the basal control diet in the afternoon feeding). During the experiment and sampling time, sheep were housed in individual pens, equipped with separate facilities for forage and concentrate, with ad libitum access to water and salt licks.

### 2.2. Sampling Procedure

All procedures on animals were approved by the Local Animal Care Ethics Committee for Animal Experiments in Warsaw (Poland, permission no. 79/2015). The samples of the rumen fluid were collected from different locations in the rumen (middle and ventral sacs) by copper tube combined with syringe by a rubber connection according to Majewska et al. [30] just before the morning feeding (0 h) and 2, 4, and 8 h after feeding for two consecutive days. At the end of the copper tube, there were numerous holes that allowed the ruminal fluid to be withdrawn from various places of the rumen. The collections were carried out with all ethical principles and respect for the animals, at their place of residence and maintenance, without exposing them to unnecessary stress. After collections, the rumen fluid was precisely mixed, filtered through a cheesecloth and stored at −18 °C until analysis. Importantly, the results obtained from samples taken within two days were pooled separately within each animal and sampling time during experimental periods (I, II, III).

### 2.3. Chemical Analysis of Animal Diet

Samples of individual diet components (meadow hay, barley, and soybean meal) were collected throughout the experiment. Briefly, samples were dried in the oven at 55 °C until they reached a constant weight, then ground, and finally passed through a sieve (1 mm). Feed samples were stored in sealed plastic containers at a room temperature until analysis. The chemical composition of animal diets was determined according to the AOAC [31], and the following procedures were applied for individual nutrients: dry matter (DM; 934.01), crude protein (954.01), crude fiber (978.10), acid detergent fiber (ADF) and acid detergent lignin (ADL; 973.18), neutral detergent fiber (NDF; 2002.04), starch (920.4), and crude ash (930.05) contents.

### 2.4. Fatty Acid Composition in Animal Diet and Rumen Fluid

Long-chain FAs in animal diet and rumen fluid samples were converted into FA methyl esters (FAME) with chloroform-methanol (2:1), subsequently saponified with KOH, esterified with SOCl_2_ (4% in methanol), and extracted with n-heptane according to the method of Folch et al. [32]. FAME samples were transferred into glass vials, diluted with n-heptane and stored at −40 °C until analysis. FA derivatives were analyzed using a gas chromatograph (Shimadzu, Tokyo, Japan), equipped with a flame-ionization detector (FID), injection port, and a capillary column (Phenomenex, BPX70; 0.25 mm i.d. × 0.25 µm film thickness × 120 m length). Helium was used as a carrier gas, with flow rate of 1 mL/min and a constant pressure of 223.4 kPa. Injector and FID temperatures were maintained at 200 °C and 240 °C, respectively; nonadecanoic acid (C19:0) served as an internal standard. The composition of FAME was analyzed using a detailed temperature gradient program described by Czauderna et al. [33] in a 1 µL sample at a split ratio of 10:1. Briefly, the analysis of the FAME profile started at 70 °C, and this temperature was maintained for 4 min. Then, the temperature was increased in several following steps: to 150 °C at a rate of 12 °C/min, to 168 °C at a rate of 8 °C/min, to 190 °C at a rate of 0.75 °C, to 210 °C at a rate of 1.8 °C/min (for 15 min), to 234 °C at a rate of 6 °C/min (for 4 min), and finally to 236 °C at a rate of 6 °C/min (for 20 min) at the end. FA peaks were identified on the basis of retention times of FAME (Supelco 37 Component FAME Mix, Bellefonte, PA, USA) and CLA *c9t11* standards (Sigma-Aldrich, Inc., St. Louis, MO, USA), and subsequently integrated using a GC software 112 (LabSolutions, GC Solution, Version 2.21, Shimadzu, Tokyo, Japan).

### 2.5. Statistical Analysis

This experiment was described as a 3 × 3 Latin square experiment design. The results are presented as mean and standard error of means (SEM). The results were subjected to repeated measures analysis of variance (ANOVA), followed by Tukey’s post-hoc test. The following main effects were considered in statistical analysis: diet (CON, OAK, TAN), sampling time (0, 2, 4, and 8 h) and their interactions. Additionally, the effect of period (I, II, and III) and animal (three sheep) were also analyzed to verify the correctness of the planned experiment. Sampling time was treated as a repeated measure factor on the same unit (within subject). The normality of data (Shapiro–Wilk test) and homogeneity of variances (Levene’s test) were tested to meet ANOVA assumptions. Data with an abnormal distribution were transformed to natural logarithms. The assumption of sphericity in repeated measures ANOVA was also checked using Mauchly’s test. Significance between means was declared at *p* ≤ 0.05, and all emerging trends were discussed at 0.05 < *p* < 0.10 (Statistica, StatSoft Polska Sp. z o.o., Cracow, Poland). The obtained data are presented in Table 2 in the form of raw data (before logarithmic transformation).

## 3. Results

The chemical composition of the diets was similar in all feeding groups (Table 1). The animals from the OAK and TAN groups received a slightly higher amount of DM as compared to the CON sheep, which was caused by the presence of the tested additives. Net tannin intake was 4 g for both sources of tannins, as planned. Intake of the remaining components was similar for all experimental groups. No refusals were observed during the experiment as the proportion of tannins in the diet was limited.

The concentration of individual FA in all diets was very similar (Table 2). As can be seen after detailed analysis, not all FAs were detected in the tested additives. A much lower concentrations of FAs were detected in the case of tannic acid, which could be expected due to the fact that it is a pure chemical compound. Compared to the oak bark extract, C18:1 *c9* and C18:2 *c9c12*, were not detected in the tannic acid. Additionally, over three times more C16:0 acid was found in oak bark extract compared to tannic acid.

In general, the animal effect did not significantly influence the results (*p* > 0.05) except for concentrations of C14:0, SA and C18:1 *c9*. However, we did not observe any significant interactions between diets and sampling time for these FA, therefore they are not discussed in detail. Moreover, there were no significant period effect.

In this study, only the results where the interaction between sampling time and diet was statistically significant are described. It was indicated that both sampling time and animal diet had a significant effect on FA profile in the rumen fluid (Table 3). The significant interactions of both experimental factors were determined for several FA, including C14:1 (*p* = 0.022), C16:1 (*p* = 0.006), C18:2 *c9c12* (*p* < 0.001), CLA *c9t11* (*p* = 0.003), C20:1 (*p* < 0.001), C20:3 n-6 (*p* < 0.001), C20:4 (*p* < 0.001), and C24:0 (*p* = 0.001). However, deeply analyzing the obtained results it can be noted that the influence of both diet and sampling time as a single effect was found only in the case of C18:2 *c9c12* and C20:3 n-6. In most cases, only significant effect of sampling time on FA concentrations in the rumen was documented. The effect of diet (as a single effect) was found only for C20:4. Interestingly, a significant interaction between two experimental factors was found for C16:1 *c9* (*p* = 0.006), while no significant effect of their action was observed when analyzed separately.

Considering the effect of the feed ration separately, the addition of TAN into diet had a significantly greater impact on FA profiles than OAK incorporation. Briefly, in the TAN group, a significantly increased LA concentration was recorded 8 h after feeding (vs. CON and OAK; *p* = 0.045), C20:3 n-6 before feeding (vs. CON; *p* = 0.021), as well as C20:4 before feeding (vs. CON and OAK; *p* = 0.001), and 8 h after feeding (vs. OAK; *p* = 0.001). The addition of OAK to the sheep diet significantly reduced C20:3 n-6 2 h after feeding (vs. CON; *p* = 0.021), as well as C20:4 8 h after feeding (vs. TAN; *p* = 0.001).

However, it is difficult to find a common relationship between the influence of time and the concentrations of individual FAs in the rumen fluid. The data demonstrated that sampling time had a heterogeneous effect. FA concentrations were mostly higher before feeding than in samples taken 2 and/or 4 h after feeding. Increased concentrations of LA in the TAN group were also observed in the rumen fluid 8 h after feeding compared to other time points. There were also exceptions to this rule, and the concentrations of certain FAs were significantly lower even 8 h after feeding (C14:1 for OAK; LA for CON).

Although the interaction between the two experimental factors for LNA was observed as a trend (*p* = 0.086), it was shown that the addition of TAN to the sheep diet significantly increased the concentration of this acid, compared to sheep receiving OAK 4 h after feeding (*p* = 0.008).

## 4. Discussion

In the present study, the introduction of sampling time allowed for the assessment of individual FA concentrations and their changes in the rumen after administration of bioactive feed ingredients. As shown in Table 3, the concentrations of FAs in the rumen fluid 2 h after feeding (and in some cases even 4 h after feeding) were significantly lower than the concentrations of FAs before feeding. This was probably related to the time during which the nutrients present in the feed (i.e., lipids) were digested with the participation of rumen microorganisms and FA were released into the environment. It could be seen that the significant impact of the TAN diet on LA and C20:4 concentrations was evident 8 h after feeding, when most of FA were released from the feed. The confirmation for our suspicions might be the reduced number of total protozoa 2 and 4 h after feeding for all feeding groups (CON, OAK, TAN) observed during preliminary analyzes, and, simultaneously, a significantly lower number of protozoa in sheep fed OAK and TAN diets (unpublished data). The reduced number of protozoa could delay the release of FA present in feed rations. Importantly, Szumacher-Strabel [35] indicates the role of protozoa, especially of the *Epidinium* genus, in the transformation of FA in the rumen. Furthermore, changes in the FA concentrations can be also a direct result of tannins action on not only microorganism consortium in general, but also on bacteria strictly involved in ruminal biohydrogenation, such as *Butyrivibrio* spp., *Fusocillus* spp., *Clostridium proteoclasticum,* and the recently revealed uncultured *Lachnospiraceae* [2,11,36,37]. Generally, tannins are considered to be microorganisms growth inhibitors [9]. However, some animals have developed several protective mechanisms to detoxify organism from high concentration of tannins. Secretion of the tannase enzyme, which breaks down hydrolysable tannins to gallic acid, is one example of such mechanisms [38]. Such protective mechanisms of microorganisms habituating rumen, may partially explain the obtained results for TAN sheep, which, despite some FA concentrations, did not significantly differ between those obtained for CON group.

Previous study showed that different types of tannins may have various effect on FA biohydrogenation [11,39]. According to the results obtained by Costa et al. [39], condensed tannins from mimosa impaired the biohydrogenation process to a greater extent than hydrolysable tannins from chestnut. Furthermore, as indicated by Vasta et al. [11], condensed tannins are considered to inhibit ruminal biohydrogenation, while hydrolysable tannins have rather modulatory effect on this process. In the current study, the TAN diet had a more pronounced effect on FAs in the rumen than the OAK diet. It was shown that only the TAN diet increased LNA and LA concentrations in the ruminal fluid, which could indicate that hydrolysable tannins present in TAN hindered FA biohydrogenation at the first stage. Unfortunately, neither additive, as expected, exerted an effect on SA concentration, despite the slightly lower concentration of this FA in the ruminal fluid of OAK sheep (Table 3). On the other hand, in the studies of Guerreiro et al. [20] and Costa et al. [21], condensed tannins from *Cistus ladanifer* extract mostly affected the first stage of biohydrogenation by stimulating the disappearance of LA and LNA without inhibition of C18:0. Despite using one type (gallotannins) and similar doses of tannins in diets, differences in the proportion of C18 FA in the rumen could also arise from the actual presence of biologically active compounds. Synthetic TAN (C_76_H_52_O_46_) was purchased from Sigma-Aldrich and, according to the specification note, it was an ester with five glucose hydroxyl groups and gallic acid acyl group. Interestingly, the main component of OAK, as reported by British Pharmaceutical Codex [40], is quercitannic acid (C_17_H_16_O_9_), which is one of the forms of TAN. In addition to the aforementioned acid, OAK also contains gallic and ellagic acids, but low levels of proanthocyanidins (condensed tannins) are also present [41]. As was mentioned above, different structural characteristics of tested supplements significantly affected the results. Furthermore, existing differences in the presence and composition of FAs in the tested additives, but not the diets (Table 2), seemed to have no impact on the obtained results.

Concerning other results obtained in the present study, negligible decreased concentration of C18:1 *c9* after OAK supplementation, differ from study assumptions, and may suggest that during biohydrogenation of this FA, some other positional isomers of *trans* C18:1 FA, including *trans-6*, *-7*, *-9*, *-10*, *-11*, *-12*, *-13*, *-14*, *-15,* and *-16*, are formed [42]. Such an effect was observed in the study by Costa et al. [39] with hydrolysable tannins from chestnut extract, and may indicate that bacteria have developed an adaptive strategy to the presence of tannins. It could be also possible that the significant effect of the animal obtained in the present study made it impossible to demonstrate statistically relevant differences between OAK and other groups for this acid. Unfortunately, in the present study we were not able to test the effect of dietary treatments on residual *trans* isomers except from C18:1 *t9*. For this FA, the significant differences were observed only for diet and time as a single effect, without their interaction effect. Similar to C18:1 *c9*, decreased concentrations of C18:1 *t9* after feeding in OAK diet (vs. CON group) were noted. 

On the other hand, an in vitro study of Carreño et al. [43] showed that OAK addition (20 g/kg DM) increased total PUFA, LNA, LA, and C18:1 *t11*, while it decreased C18:1 *t10* and SA concentrations, without any negative effects on ruminal fermentation. These results were partially confirmed in the present study, but only for the TAN diet. Perhaps the amount of OAK added to the sheep diet was too low (13 g) to have a similar effect to that observed in the abovementioned study.

The CLA *c9t11* isomer is one of 28 CLA isomers with the highest proportion and biological activity. Literature data indicate health-promoting properties of CLA *c9t11*, including reduced incidence of arteriosclerosis, diabetes, and neoplastic diseases [44]. Unfortunately, the amount of OAK and TAN additives used in the present study was insufficient to increase CLA *c9t11* concentration in the ruminal fluid, contrary to assumptions. Similar observations were made by Al-Jumaili et al. in an in vitro study [45], where incubation of the goat ruminal fluid with TAN did not significantly affect CLA *c9t11* concentration. Furthermore, various sources of condensed tannins did not affect the concentration of CLA isomers or the activity of linoleic acid isomerase, as presented in an in vitro study of Vasta et al. [18]. Another possible reason for lack of the differences in CLA concentration could be a reduced number of total protozoa in sheep receiving OAK and TAN diets (preliminary observations). Importantly, the study of Francesco et al. [46] reported that protozoa were positively associated with C18:1 *t11* and CLA *c9t11* concentrations in the rumen. It appears that protozoa do not synthesize these acids, and their presence in protozoan cells may be caused by the ingestion of bacteria. Nevertheless, FAs of protozoan origin represent a significant proportion of the entire pool flowing from the rumen [47]. Due to the fact that the present study is preliminary, it would be worth deeply examining this issue, as well as determining the number of bacteria involved in the biohydrogenation of FA in the rumen, especially *Butyrivibrio fibrisolvens* and *B. proteoclasticus,* responsible for CLA *c9t11* and SA production, respectively [16]. This would provide a more complete insight of the effect of tested additives on lipid metabolism in the rumen and clear up any appearing ambiguities in the interpretation of the obtained results.

TAN diet seemed to exert a beneficial effect on the FA profile in the ruminal fluid by increasing PUFA concentrations (including LA, LNA, C20:3 n-6, and C20:4). Our findings are consistent with studies on lambs [12,48] and kids [13], where increased PUFA concentrations were reported after the incorporation of tannins into diet. Importantly, the knowledge about the impact of bioactive compounds on rumen function (including microorganism numbers and carbohydrate fermentation) is crucial for directional increase of health-promoting properties of animal products [49]. Interestingly, our preliminary observations showed that the tested additives did not negatively affect ruminal parameters, including concentration of total short-chain FAs as end products of carbohydrate degradation in the rumen.

The results of the present study may be used as a basis for further studies on a larger group of ruminants on the effect of tannins on the improving quality and composition of FA in products of ruminant origin and consequently human health. Secondly, a larger group of animals is required for this type of research to confirm the obtained relationships and to exclude possible individual effects. In the future, an increased proportion of fat added into the diet would be also extremely useful. This is of particular importance when studying the influence of dietary factors on the process of FA biohydrogenation, as it may result in a more visible effect of OAK and TAN diet on FA in the rumen. In the present study, the fat content in the diet was about 2.45%. This value could be not enough to observe any evident changes in the quantity of FA, especially for CLA *c9t11*. It seems that apart from the dose and type of additives used in the experiment and the concentration of bioactive substances, fat content in the diet could be one of the factors, for which the impact of OAK on FA composition has not been documented in the current study or this effect was relatively small.

## 5. Conclusions

We demonstrated that the TAN diet had a greater influence on FA profile in the ruminal fluid than the OAK diet. Differences in the effects of the tested additives could also result from the presence of various types of tannins in the OAK. Additionally, an increased concentrations of both LA and LNA in the rumen may indicate that hydrolysable tannins present in TAN inhibited the initial stage of FA biohydrogenation in the rumen.

However, it is recommended to carry out further studies on a larger number of animals using higher additive doses, additionally expanded by an accurate microbiological examination in order to understand in detail the mechanism of hydrolysable tannin action on the process of FA biohydrogenation in the rumen. Targeted application of such bioactive substances will allow to increase the amount of health-promoting FA exiting the rumen to further parts of the digestive tract.

## Figures and Tables

**Table 1:** Composition of sheep diets ^1^.

Item	CON	OAK	TAN
Components (g/kg DM)			
Meadow hay	588	581	586
Barley meal	294	290	293
Soybean meal	98.0	96.8	97.7
Polfamix ^2^	19.6	19.4	19.5
Oak bark extract	-	12.6	-
Tannic acid	-	-	3.91
Chemical composition (g/kg DM)			
Dry matter	887	887	887
Crude protein ^3^	110	109	109
Crude fat	21.8	21.8	21.7
Starch	222	219	221
NDF	442	436	440
ADF	371	366	369
ADL	35.7	35.4	35.6
Crude ash	34.4	34.2	34.3
Nutrient intake (g/d)			
Dry matter	905	917	908
Crude protein	112	112	112
Crude fat	22.2	22.5	22.2
Starch	226	226	226
NDF	451	451	451
ADF	378	378	378
ADL	36.4	36.5	36.4
Crude ash	35.1	35.3	35.1

CON, control diet; OAK, diet with oak bark extract addition; TAN, diet with tannic acid addition; DM, dry matter; NDF, neutral detergent fiber; ADF, acid detergent fiber; ADL, acid detergent lignin. ^1^ diet formulated according to the recommendations for ruminants [34]; ^2^ Polfamix O-K (Trouw Nutrition Polska, Grodzisk Mazowiecki, Poland) in kilograms: g: Ca 240, Na 60, P 120, Mg 65, Zn 2.5, Mn 3.0, vit. E 1.5, Se 0.003, Co 0.015; IU: vit. A 300.000, vit. D_3_ 30.000; ^3^ expressed as N × 6.25.

**Table 2:** Fatty acid composition of the additive and diet (mg FAME/kg).

Item	CON	OAK	TAN
FA composition (mg FAME/kg additive) ^1^			
C14:0	-	25.4	15.8
C16:0	-	400	117
C16:1 *c9*	-	-	-
C18:0	-	228	201
C18:1 *c9*	-	111	-
C18:2 *c9c12*	-	439	-
C18:3 *c9c12c15*	-	-	-
C20:0	-	-	-
C20:3 n-6	-	-	-
C24:0	-	-	-
Total FA	-	1203	334
FA composition (mg FAME/kg diet) ^2^			
C14:0	33.8	34.1	33.9
C16:0	728	733	728
C16:1 *c9*	6.99	6.99	6.99
C18:0	239	242	240
C18:1 *c9*	107	109	107
C18:2 *c9c12*	739	744	739
C18:3 *c9c12c15*	694	694	694
C20:0	11.8	11.8	11.8
C20:3 n-6	13.3	13.3	13.3
C24:0	4.73	4.73	4.73
Total FA	2 578	2593	2579

CON, control diet; OAK, diet with oak bark extract addition; TAN, diet with tannic acid addition. ^1^ FA composition calculated only per kilogram of additive (tannic acid or oak bark extract); ^2^ The amounts of the individual FAs concentrations in the diets were calculated according to their content in each component and animal diet composition.

**Table 3:** Fatty acid composition of the ruminal fluid in sheep (mg FAME/100 g).

Fatty Acid	Diet (D)	Sampling Time (T)				SEM	*p*-Value		
		0 h	2 h	4 h	8 h		T	D	T × D
C14:0	CON	4.40	3.37	2.96	3.10	0.121	<0.001	0.798	0.556
	OAK	3.77	3.03	3.14	2.84				
	TAN	3.90	3.09	2.64	3.09				
C14:1	CON	3.18	2.47	2.97	3.17	0.096	<0.001	0.563	0.022
	OAK	3.64 ^a^	2.09 ^b^	2.31 ^b^	2.52 ^b^				
	TAN	3.05	2.33	2.47	3.00				
C16:0	CON	52.4	41.7	45.4	48.7	0.04	0.056	0.414	0.928
	OAK	46.6	34.3	34.8	45.2				
	TAN	52.2	43.8	48.1	47.8				
C16:1	CON	1.42	1.32	0.57	0.92	0.044	0.098	0.809	0.006
	OAK	1.14	0.80	1.09	0.80				
	TAN	1.78	1.74	1.03	1.10				
C18:0	CON	79.2	58.9	65.4	69.5	2.36	0.056	0.538	0.949
	OAK	70.8	48.6	52.3	66.4				
	TAN	77.0	51.8	57.2	61.8				
C18:1 *t9*	CON	6.57	5.31	5.83	6.16	0.150	<0.001	<0.001	0.734
	OAK	5.51	3.97	4.10	4.65				
	TAN	5.95	4.38	4.45	4.91				
C18:1 *c9*	CON	12.8	12.4	10.9	9.30	0.464	0.225	0.564	0.215
	OAK	9.70	8.98	8.93	11.5				
	TAN	13.5	12.2	10.9	10.3				
C18:2 *c9c12*	CON	10.9 ^a,b^	10.8 ^a,b^	14.9 ^a^	9.42 ^b,x^	0.050	<0.001	0.045	<0.001
	OAK	8.37	9.84	12.0	10.8 ^x^				
	TAN	9.76 ^a^	12.1 ^a^	14.1 ^a^	23.4 ^b,y^				
CLA *c9t11*	CON	0.88	1.00	0.96	0.92	0.056	0.001	0.223	0.003
	OAK	1.46 ^a^	0.42 ^b^	0.74 ^b^	0.88 ^a,b^				
	TAN	1.22 ^a,b^	0.90 ^a^	0.59 ^a,b^	1.35 ^b^				
C18:3 *c9c12c15*	CON	1.50	1.63	1.45 ^x,y^	1.30	0.063	0.325	0.008	0.086
	OAK	1.59	1.02	1.13^x^	1.48				
	TAN	1.78	1.53	2.05 ^y^	2.01				
C20:0	CON	1.00	0.63	0.82	0.68	0.032	0.201	0.844	0.889
	OAK	0.97	0.65	0.70	0.90				
	TAN	1.39	0.62	0.74	0.82				
C20:1	CON	0.95 ^a^	0.49 ^b^	0.76 ^a,b^	0.86 ^a^	0.035	<0.001	0.134	<0.001
	OAK	0.68	0.77	0.94	0.95				
	TAN	1.04 ^a^	0.47 ^b^	0.57 ^b,c^	0.83 ^a,c^				
C20:3 n-6	CON	0.45 ^a,x^	0.84 ^b,x^	0.36 ^a^	0.54 ^a^	0.018	0.002	0.021	<0.001
	OAK	0.63 ^a,b,x,y^	0.40 ^a,y^	0.52 ^a,b^	0.76 ^b^				
	TAN	0.87 ^a,y^	0.54 ^b,y^	0.56 ^b^	0.60 ^ab^				
C20:4	CON	0.68 ^x^	0.39	0.60	0.73 ^x,y^	0.036	0.668	0.001	<0.001
	OAK	0.54 ^x^	1.00	0.90	0.62 ^x^				
	TAN	0.96 ^y^	0.89	0.70	1.01 ^y^				
C24:0	CON	0.92 ^a,b^	0.61 ^a^	0.65 ^a^	0.99 ^b^	0.032	0.013	0.449	0.001
	OAK	0.81	1.13	0.95	0.93				
	TAN	0.83	1.03	0.83	1.04				
Total FA	CON	177	142	155	156	4.5	<0.001	0.441	0.904
	OAK	156	117	125	151				
	TAN	175	137	147	163				

CON, control diet; OAK, diet with oak bark extract addition; TAN, diet with tannic acid addition; SEM, standard error of mean; FAME, fatty acid methyl esters. Different letters in a row (^a,b,c^ *p* ≤ 0.05) show differences between sampling time (0, 2, 4, 8 h). Different letters in a column (^x,y^ *p* ≤ 0.05) show differences between diet (CON, OAK, TAN).

## Data Availability

The data presented in this study are available on request from the corresponding author.

## References

[1] Mueller-Harvey I. (2001). Analysis of hydrolysable tannins. Anim. Feed Sci. Technol..

[2] Patra A.K., Saxena J. (2011). Exploitation of dietary tannins to improve rumen metabolism and ruminant nutrition. J. Sci. Food Agric..

[3] Majewska M.P., Pająk J.J., Skomiał J., Miltko R., Kowalik B. (2017). The effect of lingonberry leaves and oak cortex addition to sheep diets on pancreatic enzymes activity. J. Anim. Feed Sci..

[4] Jones G.A., McAllister T.A., Muir A.D., Cheng K.J. (1994). Effects of sainfoin (*Onobrychis viciifolia* Scop.) condensed tannins on growth and proteolysis by four strains of ruminal bacteria. Appl. Environ. Microbiol..

[5] Min B.R., Solaiman S. (2018). Comparative aspects of plant tannins on digestive physiology, nutrition and microbial community changes in sheep and goats: A review. J. Anim. Physiol. Anim. Nutr..

[6] Barbehenn R.V., Constabel C.P. (2011). Tannins in plant-herbivore interactions. Phytochemistry.

[7] Makkar H.P.S. (2003). Review. Effects and fate of tannins in ruminant animals, adaptation to tannins, and strategies to overcome detrimental effects of feeding tannin-rich feeds. Small Rumin. Res..

[8] Lotfi R. (2020). A commentary on methodological aspects of hydrolysable tannins metabolism in ruminant: A perspective view. Lett. Appl. Microbiol..

[9] McSweeney C.S., Palmer B., McNeill D.M., Krause D.O. (2001). Microbial interactions with tannins: Nutritional consequences for ruminants. Anim. Feed Sci. Technol..

[10] Patra A.K., Saxena J. (2009). Dietary phytochemicals as rumen modifiers: A review of the effects on microbial population. Antonie Leeuwenhoek.

[11] Vasta V., Daghio M., Cappuci A., Buccioni A., Serra A., Viti C., Mele M. (2019). Invited review: Plant polyphenols and rumen microbiota responsible for fatty acid biohydrogenation, fiber digestion, and methane emission: Experimental evidence and methodological approaches. J. Dairy Sci..

[12] Priolo A., Bella M., Lanza M., Galofaro V., Biondi L., Barbagallo D., Ben Salem H., Pennisi P. (2005). Carcass and meat quality of lambs fed fresh sulla (*Hedysarum coronarium* L.) with or without polyethylene glycol or concentrate. Small Rumin. Res..

[13] Rana M.S., Tyagi A., Hossain S.A., Tyagi A.K. (2012). Effect of tanniniferous *Terminalia chebula* extract on rumen biohydrogenation, ∆^9^-desaturase activity, CLA content and fatty acid composition in longissimus dorsi muscle of kids. Meat Sci..

[14] Frutos P., Hervás G., Natalello A., Luciano G., Fondevila M., Priolo A. (2020). Ability of tannins to modulate ruminal lipid metabolism and milk and meat fatty acid profiles. Anim. Feed Sci. Technol..

[15] Vasta V., Bessa R.J.B., Patra A.K. (2012). Manipulating Ruminal Biohydrogenation by the Use of Plants Bioactive Compounds. Dietary Phytochemicals and Microbes.

[16] Morales R., Ungerfeld E.M. (2015). Use of tannins to improve fatty acids profile of meat and milk quality in ruminants: A review. Chilean J. Agric. Res..

[17] Manso T., Bodas R., Castro T., Jimeno V., Mantecon A.R. (2008). Animal performance and fatty acid composition of lambs fed with different vegetable oils. Meat Sci..

[18] Vasta V., Makkar H.P.S., Mele M., Priolo A. (2009). Ruminal biohydrogenation as affected by tannins in vitro. Br. J. Nutr..

[19] Khiaosa-Ard R., Bryner S.F., Scheeder M.R.L., Wettstein H.-R., Kreuzer M., Soliva C.R. (2009). Evidence for the inhibition of the terminal step of ruminal α-linolenic acid biohydrogenation by condensed tannins. J. Dairy Sci..

[20] Guerreiro O., Alves S.P., Costa M., Cabo Â., Duarte M.F., Jerónimo E., Bessa R.J.B. (2016). Effects of extracts obtained from *Cistus ladanifer* L. on in vitro rumen biohydrogenation. Anim. Feed Sci. Technol..

[21] Costa M., Alves S.P., Cabo Â., Guerreiro O., Stilwell G., Dentinho M.T., Bessa R.J.B. (2017). Modulation of in vitro rumen biohydrogenation by *Cistus ladanifer* tannins compared with other tannins sources. J. Sci. Food Agric..

[22] Elansary H.O., Szopa A., Kubica P., Ekiert H., Mattar M.A., Al-Yafrasi M.A., El-Ansary D.O., El-Abedin T.K.Z., Yessoufou K. (2019). Polyphenol profile and pharmaceutical potential of *Quercus* spp. bark extracts. Plants.

[23] Duda-Chodak A., Tarko T., Rus M. (2011). Antioxidant activity and total polyphenol content of selected herbal medicine products used in Poland. Herb. Pol..

[24] Majewska M.P., Kowalik B. (2019). Growth performance, carcass characteristics, fatty acid composition, and blood biochemical parameters of lamb fed diet with the addition of lingonberry leaves and oak bark. Eur. J. Lipid Sci. Technol..

[25] Majewska M.P., Miltko R., Bełżecki G., Kędzierska A., Kowalik B. (2021). Protozoa population and carbohydrate fermentation in sheep fed diet with different plant additives. Anim. Biosci..

[26] Singh B., Bhat T.K., Sharma O.P. (2001). Biodegradation of tannic acid in an in vitro ruminal system. Livest. Prod. Sci..

[27] Silanikove N., Perevolotsky A., Provenza F.D. (2001). Use of tannin-binding chemicals to assay for tannins and their negative postingestive effects in ruminants. Anim. Feed Sci. Technol..

[28] Peréz V., Doce R.R., García-Pariente C., Hervás G., Ferrera M.C., Mantecón Á.R., Frutos P. (2011). Oak leaf (*Quercus pyrenaica*) poisoning in cattle. Res. Vet. Sci..

[29] Rojas-Román L.A., Castro-Pérez B.I., Estrada-Angulo A., Angulo-Montoya C., Yocupicio-Rocha J.A., López-Soto M.A., Barreras A., Zinn R.A., Plascencia A. (2017). Influence of long-term supplementation of tannins on growth performance, dietary net energy and carcass characteristics: Finishing lambs. Small Rumin. Res..

[30] Majewska M.P., Miltko R., Bełżecki G., Skomiał J., Kowalik B. (2017). Supplementation of rapeseed and linseed oils to sheep rations: Effects on ruminal fermentation characteristics and protozoal populations. Czech J. Anim. Sci..

[31] AOAC (2011). Association of Official Analytical Chemists, Official Methods of Analysis.

[32] Folch J., Lees M., Sloane Stanley G.H. (1957). A simple method for the isolation and purification of total lipids from animal tissues. J. Biol. Chem..

[33] Czauderna M., Kowalczyk J., Krajewska K.A., Rozbicka A.J., Michalski J.P. (2009). Dietary selenite and conjugated linoleic acid isomers influence fatty acid concentrations in the liver and femoral muscles of rats. J. Anim. Feed Sci..

[34] Strzetelski J., IZ PIB-INRA (2009). Normy żywienia przeżuwaczy. Wartość pokarmowa francuskich i krajowych pasz dla przeżuwaczy (in Polish).

[35] Szumacher-Strabel M. (2010). The role of animal feeding in obtaining animal products rich in health promoting agents. Kosmos.

[36] Vasta V., Yáñez-Ruiz D.R., Mele M., Serra A., Luciano G., Lanza M., Biondi L., Priolo A. (2010). Bacterial and protozoa communities and fatty acid profile in the rumen of sheep fed a diet containing added tannins. Appl. Environ. Microbiol..

[37] Buccioni A., Pauselli M., Viti C., Minieri S., Pallara G., Roscini V., Rapaccini S., Trabalza Marinucci M., Lupi P., Conte G. (2015). Milk fatty acid composition, rumen microbial population and animal performances in response to diets rich in linoleic acid supplemented with chestnut or quebracho tannins in dairy ewes. J. Dairy Sci..

[38] Skene I.K., Brooker J.D. (1995). Characterization of tannin acylhydrolase activity in the ruminal bacterium *Selenomonas Ruminantium*. Anaerobe.

[39] Costa M., Alves S.P., Cappucci A., Cook S.R., Duarte A., Caldeira R.M., McAllister T.A., Bessa R.J.B. (2018). Effects of condensed and hydrolyzable tannins on rumen metabolism with emphasis on the biohydrogenation of unsaturated fatty acids. J. Agric. Food Chem..

[40] British Pharmaceutical Codex (1911). An Imperial Dispensatory for the Use of Medical Practitioners and Pharmacists.

[41] Smeriglio A., Barreca D., Bellocco E., Trombetta D. (2017). Proanthocyanidins and hydrolysable tannins: Occurrence, dietary intake and pharmacological effects. Brit. J. Pharmacol..

[42] Mosley E.M., Powell G.L., Riley M.B., Jenkins T.C. (2002). Microbial biohydrogenation of oleic acid to *trans* isomers in vitro. J. Lipid Res..

[43] Carreño D., Hervás G., Toral P.G., Belenguer A., Frutos P. (2015). Ability of different types and doses of tannin extracts to modulate in vitro ruminal biohydrogenation in sheep. Anim. Feed Sci. Technol..

[44] Tanaka K. (2005). Occurrence of conjugated linoleic acid in ruminant products and its physiological functions. Anim. Sci. J..

[45] Al-Jumaili W.S., Goh Y.M., Jafari S., Rajion M.A., Jahromi M.F., Ebrahimi M. (2017). An in vitro study on the ability of tannic acid to inhibit methanogenesis and biohydrogenation of C18 PUFA in the rumen of goats. Ann. Anim. Sci..

[46] Francisco A.E., Santos-Silva J.M., Portugal A.P.V., Alves S.P., Bessa R.J.B. (2019). Relationship between rumen ciliate protozoa and biohydrogenation fatty acid profile in rumen and meat of lambs. PLoS ONE.

[47] Lourenҫo M., Ramos-Morales E., Wallace R.J. (2010). The role of microbes in rumen lipolysis and biohydrogenation and their manipulation. Animal.

[48] Cabiddu A., Molle G., Decandia M., Spada S., Fiori M., Piredda G., Addis M. (2009). Responses to condensed tannins of flowering sulla (*Hedysarum coronarium* L.) grazed by dairy sheep. Part 2: Effects on milk fatty acid profile. Livest. Sci..

[49] Vahmani P., Ponnampalam E.N., Kraft J., Mapiye C., Bermingham E.N., Watkins P.J., Proctor S.D., Dugan M.E.R. (2020). Bioactivity and health effects of ruminant meat lipids. Invited Review. Meat Sci..

